# The Rationale of Neprilysin Inhibition in Prevention of Myocardial Ischemia-Reperfusion Injury during ST-Elevation Myocardial Infarction

**DOI:** 10.3390/cells9092134

**Published:** 2020-09-21

**Authors:** Alessandro Bellis, Ciro Mauro, Emanuele Barbato, Giuseppe Di Gioia, Daniela Sorriento, Bruno Trimarco, Carmine Morisco

**Affiliations:** 1 Dipartimento di Scienze Biomediche Avanzate, Università FEDERICO II, 80131 Napoli, Italy; abellis82@vodafone.it (A.B.); emanuele.barbato@unina.it (E.B.); di-gioia@libero.it (G.D.G.); daniela.sorriento@unina.it (D.S.); trimarco@unina.it (B.T.); 2 Unità Operativa Complessa Cardiologia con UTIC ed Emodinamica—Dipartimento Emergenza Accettazione, Azienda Ospedaliera “Antonio Cardarelli”, 80131 Napoli, Italy; ciro.mauro1957@gmail.com; 3 Cardiac Catheterization Laboratory, Montevergine Clinic, 83013 Mercogliano (AV), Italy

**Keywords:** neprilysin, myocardial ischemia-reperfusion injury, natriuretic peptide, angiotensin II, bradykinin, apelin, substance P, adrenomedullin, sacubitril/valsartan

## Abstract

During the last three decades, timely myocardial reperfusion using either thrombolytic therapy or primary percutaneous intervention (pPCI) has allowed amazing improvements in outcomes with a more than halving in 1-year ST-elevation myocardial infarction (STEMI) mortality. However, mortality and left ventricle (LV) remodeling remain substantial in these patients. As such, novel therapeutic interventions are required to reduce myocardial infarction size, preserve LV systolic function, and improve survival in reperfused-STEMI patients. Myocardial ischemia-reperfusion injury (MIRI) prevention represents the main goal to reach in order to reduce STEMI mortality. There is currently no effective therapy for MIRI prevention in STEMI patients. A significant reason for the weak and inconsistent results obtained in this field may be the presence of multiple, partially redundant, mechanisms of cell death during ischemia-reperfusion, whose relative importance may depend on the conditions. Therefore, it is always more recognized that it is important to consider a “multi-targeted cardioprotective therapy”, defined as an additive or synergistic cardioprotective agents or interventions directed to distinct targets with different timing of application (before, during, or after pPCI). Given that some neprilysin (NEP) substrates (natriuretic peptides, angiotensin II, bradykinin, apelins, substance P, and adrenomedullin) exert a cardioprotective effect against ischemia-reperfusion injury, it is conceivable that antagonism of proteolytic activity by this enzyme may be considered in a multi-targeted strategy for MIRI prevention. In this review, by starting from main pathophysiological mechanisms promoting MIRI, we discuss cardioprotective effects of NEP substrates and the potential benefit of NEP pharmacological inhibition in MIRI prevention.

## 1. Introduction

The improvement of strategies for the protection of myocardium following acute ischemic insult represents, nowadays, the real challenge of the clinical research in cardiology. In fact, despite the widespread use of the best available therapies (i.e., timely revascularization, antiplatelet and anti-remodeling drugs, vascular devices), up to 30% of patients with ST-elevated myocardial infarction (STEMI) still develop left ventricular remodeling (LVR) [1,2,3,4,5] and heart failure (HF) [6,7].

The reduction of the infarction area and the preservation of myocardial metabolism represent the pathophysiological basis of cardioprotection. The extension of the resulting infarction depends on (1) the size of the ischemic area at risk, (2) the duration and intermittency of coronary occlusion, (3) the magnitude of residual collateral blood flow, and (4) the extent of coronary microvascular dysfunction. In humans, 30–50% of the area at risk is still viable, and therefore, salvageable by reperfusion, after 4–6 h from the onset of anginal symptoms, as estimated from magnetic resonance imaging and biomarker analysis of myocardial salvage [8]. Even after 12 h of coronary occlusion, there is viable myocardium, and interventional reperfusion can limit infarct size (IS) [9].

Whereas reperfusion is mandatory to salvage ischemic myocardium from impending infarction, it also inflicts a metabolic injury that is not only reversible, such as stunning [10,11,12], but also irreversible and manifest, such as increased IS and microvascular dysfunction. This phenomenon is called myocardial ischemia-reperfusion injury (MIRI). Thus, efficient cardioprotection is the result of early myocardial reperfusion (mechanical by the primary percutaneous coronary intervention [pPCI] or pharmacological by fibrinolysis) and MIRI prevention. This latter may be induced by different endogenous compounds, such as natriuretic peptides (NPs), angiotensin (Ang), opioids, adenosine (ADE), bradykinin (Bk), apelin, substance P (SP), and adrenomedullin (ADM). Interestingly, most of these molecules (NPs, Ang, Bk, apelin, SP, and ADM) are substrates of aminopeptidase neprilysin (NEP). Furthermore, reduced expression of NEP has been found in heart regions at 24 h since exposure to brief repeated periods of ischemia-reperfusion (late ischemic preconditioning; late PC), raising the concern that functional down-regulation of this enzyme plays a pivotal role in MIRI prevention by late PC [13].

Therefore, by starting from MIRI pathophysiology, this review focuses on cardioprotective pathways activated by NEP substrates and the clinical role of NEP inhibition in the prevention of MIRI.

## 2. Pathophysiology of Myocardial and Coronary Microvascular Reperfusion Injury

The etiology of MIRI is multifactorial, and several molecular mechanisms have been shown to be involved, including reactive oxygen species (ROS) production, defects in calcium handling, mitochondrial damage, and inflammation [14]. Indeed, excessive oxygen-free radical production leads to structural damage and functional or metabolic disorder. Inadequate re-synthesis of ATP, loss of membrane phospholipids, and intracellular calcium overload also contribute to MIRI [15,16]. Here, we discuss the main pathophysiological mechanisms promoting MIRI.

### 2.1. Apoptosis

Oxidative stress and calcium overload can both induce the abrupt opening of the mitochondrial permeability transition pores (MPTPs) during MIRI [17,18]. The abrupt opening of MPTPs causes mitochondrial swelling and uncoupling of oxidative phosphorylation, thereby inducing apoptosis and cell necrosis [14,19,20]. Apoptosis is an energy-dependent process. It gets activated during ischemia, but it is not affected until reperfusion (when oxygen supply is reinitiated). Caspase activation is a major event in apoptosis. It has been demonstrated that pan-caspase inhibition during early reperfusion protects the myocardium against MIRI in a murine model [21]. Consistently, specific inhibition of individual caspases (such as caspase-3) also mitigates MIRI in a porcine model [22].

### 2.2. Autophagy and Mitophagy

Activation of cellular autophagy and degradation of damaged mitochondria by lysosomes (a phenomenon called “mitophagy”) is generally beneficial because they reduce MIRI [23]. As an example, the autophagy activator chloramphenicol reduces IS in a pig model [24]. Furthermore, mitophagy activation via Ulk1/Rab9/Rip1/Drp1 protects the heart against MIRI [25]. However, the role of autophagy still needs deeper dissection. It is well-known that ischemia stimulates autophagy through an adenosine monophosphate-activated protein kinase (AMPK)-dependent mechanism, whereas ischemia-reperfusion stimulates autophagic myocardial cell death and left ventricular dysfunction through a Beclin 1–dependent but AMPK-independent mechanism [26]. This last event is associated with an increase of autophagosomes containing damaged mitochondria [27]. Likewise, the down-regulation of autophagy decreases the number of apoptotic cardiomyocytes in MIRI [28]. Thus, autophagy may be protective during ischemia, whereas it may be detrimental during reperfusion [26]. Therefore, autophagy exerts a key role in the pathogenesis of MIRI [29] and represents a potential target for molecular therapeutic strategy.

### 2.3. Defects in Calcium Handling

The common clinical characteristics of MIRI are reperfusion arrhythmias [30], myocardial stunning [31], IS enlargement [32], microvascular obstruction (MVO) [12], and intramyocardial hemorrhage (IMH) [33]. In particular, perturbations of calcium homeostasis play an important role in reperfusion arrhythmias, including idioventricular rhythm, ventricular tachycardia, and ventricular fibrillation after MIRI [34]. The inhibition of intracellular Ca^2+^ overload improves the recovery of heart function and prevents the heart from contracture during reperfusion [35]. Consistently, damage to the cell membrane, which causes dysregulation of Na^+^/Ca^2+^ exchange and Na^+^/H^+^ exchange, is one of the reasons for cell edema leading to myocardial stunning [36].

### 2.4. Inflammation

Inflammation exerts a key role in cardiovascular diseases, as extensively reported in the literature [37,38]. Inflammatory responses exert a different effect in the acute and chronic phase. The acute activation of inflammatory responses, including inflammatory cells infiltration in the myocardium and cytokines release, contributes to the healing process, whereas prolonged activation triggers pro-apoptotic pathways [39]. In particular, emerging evidence suggests a potential role of neutrophil extracellular traps (NETs) in linking prolonged sterile inflammation and resistant thrombosis. NETs are a weblike structure composed of decondensed chromatin (DNA backbone) released from neutrophils in response to activated platelets [40], activated endothelial cells [41], and proinflammatory cytokines [42]. Externalized nucleosomes, in conjunction with neutrophil elastase and cathepsin G, can induce intravascular thrombus formation, thereby contributing to the pathogenesis of MIRI. Interestingly, an experimental study showed the long-term benefit of a novel deoxyribonuclease I (the enzyme implied in DNA degradation)-based reperfusion strategy in a murine model of MIRI [43].

In addition, there is a growing body of evidence raising the hypothesis that the genetically-driven interaction between inflammation and thrombosis plays a pivotal role not only in the etiology of myocardial infarction (MI) [44,45], but also in the coronary microvascular ischemia-reperfusion injury (IRI).

### 2.5. Cross-Talk between MIRI and Coronary Microvascular IRI

The coronary circulation with atherosclerotic plaque rupture and superimposed thrombosis is not only the culprit of myocardial ischemia but, after the restoration of coronary blood flow, it is also the target of IRI [11,46]. IRI in the coronary circulation manifests as microvascular dysfunction, primarily by increased capillary permeability and edema [47,48], coronary microembolization of atherosclerotic particular debris [49], impaired vasomotion secondary to endothelial and vascular smooth muscle damage [50,51,52], and release of vasoconstrictor substances from the atherosclerotic lesion [53,54,55], stasis due to platelet, leukocyte and erythrocyte aggregates in the microcirculation [56,57,58], and finally, capillary destruction and hemorrhage [59,60]. Impaired myocardial blood flow despite restoration of epicardial coronary patency was first reported by Krug et al. [33], and Kloner et al. subsequently characterized the no-reflow phenomenon (NR) as the most severe form of coronary microvascular IRI [61]. NR is observed in ~35% of patients after STEMI [62]. The incidence of NR increases with the delay to reperfusion [63], and both, NR and IMH, are powerful and clinically important adverse prognostic factors [64,65,66,67]. The causal relationship between myocardial and coronary microvascular IRI is largely unclear, although they are closely associated [68,69]. Possibly, there is a common pathophysiologic mechanism, such as the formation of ROS [70], that underlies myocardial and coronary microvascular IRI, but with a distinct impact on both compartments.

### 2.6. Cardioprotective Molecular Mechanisms: RISK and SAFE Pathways

Two main cardioprotective molecular pathways have been described [14,20,71]. The reperfusion injury salvage kinase (RISK) pathway has been postulated to be a combination of two parallel cascades, phosphatidyl-inositol-3 kinase (PI3K)-protein kinase B (Akt) and MEK1-ERK1/2. The authors of this term suggest that it refers to a group of pro-survival protein kinases, which confer cardioprotection when activated specifically at the time of reperfusion [72]. It has been suggested that some exogenous cardioprotective agonists activate PI3K, Akt, and ERK at the early reperfusion stage of IRI. It has been reported that the RISK pathway is involved in PC [73]. After PC cycles, PI3K/Akt/glycogen synthase kinase 3β (GSK 3β) cascade is activated together with ERK with consequent transactivation of the epidermal growth factor receptor. GSK 3β is suggested to be the downstream point of convergence of the RISK pathway. GSK 3β, when phosphorylated, inhibits MPTPs opening, which is cardioprotective. Thus, there is an idea that RISK activation at early reperfusion is a unifying pattern of cardioprotective signaling [74,75]. However, there are some reports that would contradict such a concept. It has been reported that GSK 3β is not required for cardioprotection induced by PC and post-conditioning (Post-C) [76] and that an intact RISK pathway is not necessarily required for Post-C in all species [77].

Survivor Activating Factor Enhancement (SAFE) has been indicated as a further cardioprotective signaling pathway. Key components of SAFE have been suggested to be cytokines of the immune system and transcription factors [78]. The crucial part of the pathway is STAT 3, a transcription factor. It has been shown that the trigger phase of PC is STAT 3-dependent [79] and that Post-C also requires activation of STAT 3 [80]. It is yet unclear whether cytosolic or mitochondrial STAT 3 are involved in mediating cardioprotection. STAT 3 seems to regulate mitochondrial respiration short term and expression of cardioprotective proteins long-term [81]. It has been postulated that STAT 3 is located in the matrix of mitochondria, where it regulates complex I respiration and inhibits MPTPs opening and ROS formation [82]. However, so far, it has been only established that STAT 3-mediated cardioprotection involves the up-regulation of proteins, while the full function in regulating mitochondrial respiration is yet to be elucidated [83,84]. The involvement of STAT 5 was suggested in remote PC [85].

Interestingly, drugs activating both pathways (such as the sphingosine-1-phosphate receptor agonist fingolimod) ameliorate MIRI and reduce MI size in a porcine model [22].

## 3. Neprilysin

NEP, an endothelial cell surface zinc metallopeptidase, is widely expressed in endothelial cells, smooth muscle cells, cardiac myocytes, renal epithelial cells, and fibroblasts. It is found in the lung, gut, adrenal glands, brain, and heart. NEP catalyzes the degradation of cardioprotective peptides, including atrial natriuretic peptide (ANP), brain natriuretic peptide (BNP), C-type natriuretic peptide (CNP), Ang II, Bk, SP, and ADM at different degrees [86]. The catalytic site of NEP, like other proteases, has a specific size, shape, and unique distribution of charges that all aid in the binding of the substrate to the catalytic site within the enzyme. The binding site within NEP does not vary, but NEP displays a different binding affinity for its substrates: high affinity for ANP, CNP, and Ang I and II, and low affinity for BNP, ET-1, and Bk [86]. More recently, it has been reported that NEP cleaves apelin peptides [87].

Few data explored the effects of NEP inhibition using endogenous substrates at various concentrations, thereby limiting our knowledge about NEP activity in specific stress conditions. However, it could be hypothesized that NEP enzymatic affinity may change based on specific substrate concentrations. Accordingly, it has been demonstrated that elevated plasma BNP is an endogenous NEP inhibitor, and it works as an important self-regulation mechanism of NPs concentration [88]. Furthermore, it has been reported that NEP may play a more dominant role in Bk degradation when levels of this peptide are high [89].

An early event in STEMI is the acute and marked increase of ventricular expression and release of ANP and BNP [90]. The time course of the plasma BNP and ANP levels could be divided into two patterns: a monophasic pattern with one peak at about 16 h after admission and a biphasic pattern with two peaks at about 16 h and 5 days after admission. There were significantly more patients with anterior infarction, congestive HF, a higher level of maximal creatine kinase-MB isoenzyme, and lower left ventricular ejection fraction in the biphasic group than in the monophasic group [90]. However, both peaks were significantly below the cut-off value for endogenous NEP inhibition [88]. Consistently, it has been demonstrated that Bk concentration decuples within 48 h since STEMI onset [91]. Therefore, it is conceivable that NEP could exert a primary catalytic role in the early phase of STEMI, when NPs plasmatic levels are not so high to inhibit its activity, and Bk significantly increases.

## 4. NEP Substrates and Their Cardioprotective Effects

**Figure 1:**
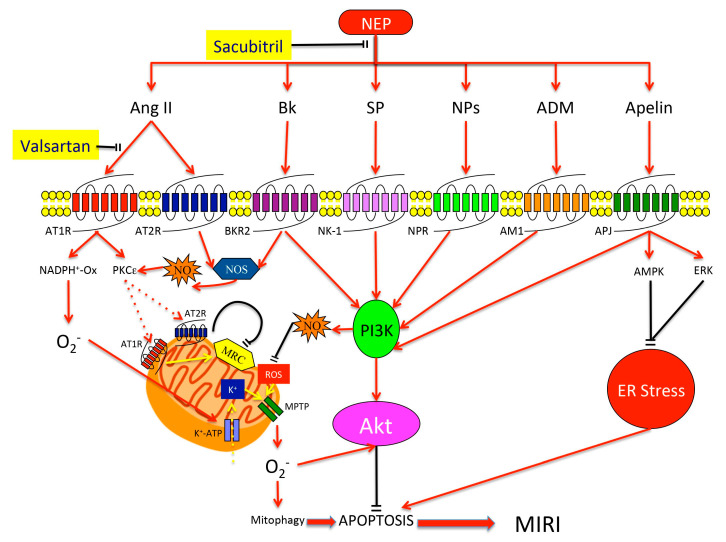
Molecular mechanisms of cardiomyocytes protection against MIRI and the potential effect of concomitant NEP inhibition/AT1R antagonism. NEP inhibition by sacubitril leads to increased levels of plasmatic Ang II, Bk, SP, NPs, ADM, and apelin. Increased Ang II levels, through a PKCε dependent mechanism, lead to the AT1R and AT2R translocation from plasmatic to mitochondrial membrane where they exert their protective role. In particular, plasmatic AT1Rs stimulate NOS activity leading to superoxide generation, mitochondrial K^+^-ATP channel activity, membrane depolarization, and MPTPs opening. This process enhances the efflux of superoxide into the cytoplasm and promotes the activation of pro-survival kinases. By contrast, plasmatic AT2R stimulation leads to NOS-NO-PKG stimulation that further stimulates PKCε. In the mitochondria, the up-regulation of AT2R/AT1R protein levels controls the MRC activity through a stimulatory mechanism composed of ROS and an inhibitory pathway involving NOS-NO. This latter exerts a master control on respiratory function as it modulates AT1Rs, while tonically suppressing electron transport chain activity. Concomitant inhibition of AT1R by valsartan blocks pro-apoptotic mechanisms mediated by this receptor, thereby, empowering pro-survival pathways induced by AT2R. Increased levels of Bk, SP, NPs, ADM, and apelin lead to PI3K-Akt and GSK-3β protective pathways activation and suppression of pro-apoptotic mechanisms induced by ER stress. These cardioprotective actions are mediated by NO synthesis and by direct inhibition of caspase-3 cleavage. MIRI, myocardial ischemia-reperfusion injury; NEP, neprilysin; AT1R and AT2R, angiotensin receptor type 1 and 2; Ang II, angiotensin II; Bk, bradykinin; SP, substance P; NPs, natriuretic peptides; ADM, adrenomedullin; PKCε, protein kinase Cε; NOX, NAD(P)H oxidase; MPTPs, mitochondrial permeability transition pores; NOS, nitric oxide synthase; NO, nitric oxide; PKG, protein kinase G; PI3K, phosphatidyl-inositol-3 kinase; ROS, reactive oxygen species; MRC, mitochondrial respiratory chain; ER, endoplasmic reticulum.

Neprilysin catalyzes the degradation of several substrates, which are promoters of peripheral vasodilation (NPs, BK, apelin, SP, and ADM) or vasoconstrictors (Ang II, Endothelin I) [86]. Here, we discuss their mechanisms of action and cardioprotective effects (Figure 1).

### 4.1. Natriuretic Peptides (NPs)

The NPs include ANP, BNP, and CNP. ANP is derived from pre-proANP, a 151 amino acid precursor, and is primarily expressed and stored in the atria, although lower concentrations can be found in the ventricles and in the kidneys. Atrial wall stress stimulates ANP secretion. Human BNP is similarly synthesized as a pre-hormone, pre-proBNP, a 134 amino acid peptide, and cleaved into proBNP, which is 108 amino acids in length. ProBNP is subsequently cleaved into the biologically active 32 amino acid BNP, plus the 76 amino acid NT-proBNP. Although low levels of BNP are found in the atria, a greater concentration of BNP is stored in the ventricles, and it is released in response to cardiac stress, such as volume overload. CNP is the most expressed NP in the brain, but it is also produced in chondrocytes and endothelial cells. After a vascular insult or endothelial injury, CNP neo-intimal expression increases [92].

NPs possess several physiological effects within the body [93,94,95,96,97]. Nevertheless, the main hormonal effects are cardiovascular ones. Indeed, ANP and BNP in the circulation cause vasodilation, increase diuresis and natriuresis, and act as counter-regulatory to the renin-angiotensin system (RAS) [95,98,99]. Speculation has been whether the cardioprotective actions of NPs are due to direct actions on the heart or indirect actions through peripheral vasodilation, and thereby a theoretically desirable decrease in pre- and after-load or both. However, clear evidence exists on direct beneficial infarct-limiting effects of NPs in the cardiomyocytes.

In vitro studies showed that synthetic ANP, via cGMP-forming trans-membrane guanylyl cyclase (GC)-receptor A (GC-A or NPR-A), reduces TNF-α-induced endothelial hyperpermeability [100] and limits neutrophil adhesion to hypoxic endothelium [101]. In vivo, in the systemic microcirculation, ANP attenuates acute, immediate inflammatory effects of mast cells or histamine [102]. These observations suggest that ANP and BNP, released in the very early phase of acute MI, might improve the endothelial barrier and attenuate the inflammatory infiltration of the myocardium, thereby limiting the area of necrosis. Furthermore, CNP from cardiomyocytes, endothelial cells, and fibroblasts coordinates and preserves the cardiac structure, function, and coronary vasoreactivity via activation of NP receptor C (NPR-C) [103].

It has been reported that cardioprotective action of NPs is mediated through mitochondrial, but not sarcolemmal, K^+^-ATP-channel opening [104,105]. Urodilatin, which is a kidney-specific form of ANP, limited acute reperfusion injury in the isolated rat heart by attenuating cGMP depletion [106]. ANP infusion in the isolated rabbit heart, given just before and during early reperfusion, limits infarct size possibly through the RISK pathway involving activation of PI3/Akt and Erk1/2 kinases [107]. Consistently, ANP and urodilatin protected cardiomyocytes subjected to anoxia/reoxygenation against reoxygenation hypercontracture [108]. It has been also demonstrated that the cardioprotective actions of urodilatin were due to inhibition of Ca^2+^-induced hypercontracture caused by an increase in sarcoplasmic reticulum (SR) ATPase (SERCA) activity [109].

A reduced release of myocardial creatinine kinase isoform MB by ~15%, an increase in left ventricular ejection fraction by ~5%, and a decrease in reperfusion injuries (defined as malignant arrhythmias, re-elevation of ST-segment, or worsening of chest pain) have been reported in a clinical placebo-controlled trial on ANP infusion during MI [110]. Finally, plasma BNP level before pPCI may be a predictor of IRI and the resultant extent of myocardial damage. In particular, high plasma BNP levels might have a clinically important protective effect on ischemic myocardium in patients with STEMI who receive pPCI [111].

### 4.2. Angiotensin (Ang)

Cardiac RAS (cRAS) plays an important role in MIRI. Ang I converts into vasoconstrictor Ang II peptide, formed of eight amino acids, via the angiotensin-converting enzyme (ACE), whereas Ang I and Ang II are converted into vasodilator peptide Ang 1–7 by ACE2 and NEP [112].

Ang II has been shown to reduce IS by affecting mitochondrial respiration and cardiac functions [113,114]. In fact, recent studies suggest that intracellular Ang II exerts protective effects in cells during high extracellular levels of the hormone or chronic stimulation of the local tissue RAS. Notably, the intracellular RAS (iRAS) described in neurons, fibroblasts, renal cells, and cardiomyocytes provided new insights into regulatory mechanisms mediated by intracellular Ang II type 1 (AT1Rs) and 2 (AT2Rs) receptors, particularly in mitochondria and nucleus. For instance, Ang II, through nuclear AT1Rs, promotes protective mechanisms through the stimulation of AT2R signaling cascade, which involves mitochondrial AT2Rs and Mas receptors [115]. The stimulation of nuclear Ang II receptors enhances mitochondrial biogenesis by peroxisome proliferator-activated receptor-γ coactivator-1α and increases sirtuins activity, thereby protecting the cell against oxidative stress [115]. Thus, despite abundant data on the deleterious effects of Ang II, a growing body of studies also supports a protective role for cRAS and iRAS that could be of relevance to support the hypothesis of NEP inhibition in MIRI prevention.

Ang 1–7 regulates cardiac functions, blood pressure, cardiac hypertrophy, HF, and the growth of cells. In particular, Ang 1–7 has anti-proliferative action on the vascular smooth muscles, cardiac muscle cells, and improves endothelial functions by releasing Bk and nitric oxide (NO) [116,117]. The ACE2 activator (diminazene aceturate) exerts a cardioprotective effect in cardiomyocytes hypertrophy [118]. Furthermore, diminazene aceturate attenuates LVR post-MI. Indeed, ACE2 activates the circulating endothelial progenitor cells in the blood that decreases the inflammatory cells and increases the cardiac progenitor cells in the region of peri-infarct cardiac muscles [119]. It also enhances the function of the heart and increases the level of angiotensin 1–7 in both humans and rats [120,121].

### 4.3. Bradykinin (Bk)

The kallikrein-kinin system has well-established cardioprotective actions. Although both plasma and tissue kallikrein (KLK1) produce kinin peptides, KLK1 plays a major role in cardioprotection. Studies of KLK1 gene knockout mice reveal an essential role for KLK1 in the maintenance of Bk levels in the heart [122]. Whereas, some of the benefits of KLK1 appear to be due to increased Bk formation [123], KLK1 may also directly activate the Bk receptor type 2 (BKR2) by a mechanism that is independent of increased Bk formation [124].

Bk is a peptide made up of nine amino acids that increase vascular permeability and acts as a potent vasodilator through the stimulation of specific endothelial BKR2. BKR2 couples to Gi proteins and activates the downstream endothelial nitric oxide synthase (eNOS)/protein kinase G (PKG) and RISK pathways, including the two parallel cascades, PI3K-Akt, and MEK1-ERK1/2, against MIRI [125]. Furthermore, Bk activates cyclo-oxygenase, and prostacyclin synthesis to attenuate IS [126,127,128].

**Figure 2:**
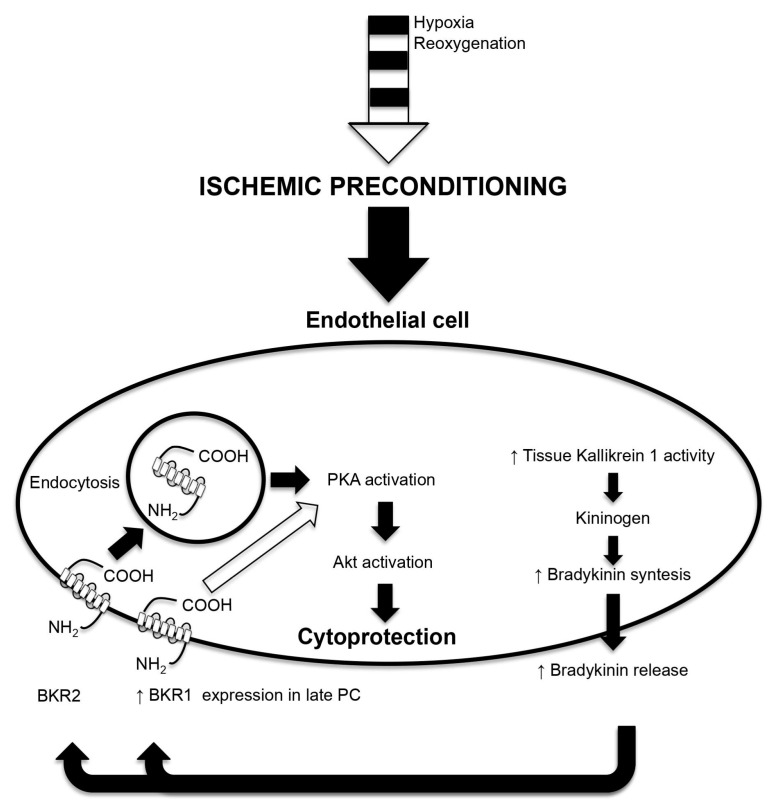
Repeated episodes of hypoxia-reoxygenation enhance the synthesis and the release in the extracellular space of bradykinin (Bk), through an increase of tissue kallikrein 1 (KLK1) activity. The released Bk by an autocrine mechanism binds its receptors. These consist of 2 types of seven transmembrane domain G protein-coupled receptors: type 2 (BKR2), which is constitutively expressed; and type 1 (BKR1), which is not constitutively expressed, but it is up-regulated by late PC. The binding of Bk to BKR2 induces the endocytosis of the receptor; this phenomenon activates protein kinase A (PKA), which, in turn, phosphorylates/activates Akt. This mechanism accounts for the cytoprotective effect of the early and late preconditioning (PC). The binding of Bk to BKR1 through the activation of PKA-Akt accounts for cytoprotection in the late window of PC.

However, Bk does not only protect the heart against MIRI, but also prevents apoptotic death induced by prolonged hypoxia in arterial endothelial cells. In particular, PC induces Bk synthesis by increasing KLK1 activity in endothelial cells [129]. Released Bk, through an autocrine mechanism, promotes cytoprotection against prolonged hypoxia [129]. This phenomenon is mainly mediated by the internalization of BKR2, which, in turn, promotes the cross-talk between protein kinase A (PKA) and Akt [129], independently by NO synthesis (Figure 2) [130]. Because these results showed that locally synthesized Bk is implied in arterial endothelium survival, they account for a systemic protective role of Bk against IRI.

Bk has a very short half-life because many different enzymes cleave it and may participate in its metabolism, and peptidase activity (ACE, NEP, NEP2, aminopeptidase, carboxypeptidase, endothelin converting enzyme) plays a major role in the tissue-specific regulation of Bk levels [131]. Therefore, it is difficult to modulate Bk concentration by blocking a single degradation pathway. However, an enzyme’s contribution to Bk degradation and the effect of inhibition of that enzyme on Bk degradation depends not only on the concentration of the enzyme, but also on the Bk concentration and the K_m_ (Michaelis constant) of each peptidase [131]. Thus, depending on an enzyme’s abundance and k_cat_ (turnover number), an enzyme with a low K_m_, such as ACE, may have a predominant role in Bk metabolism when Bk levels are low, whereas an enzyme with a higher Km (e.g., carboxypeptidase and NEP) may play a more dominant role when Bk levels are high [89], such as in STEMI [91,132].

Furthermore, the effect of an enzyme inhibition on Bk levels depends not only on the specific enzyme’s contribution to Bk metabolism relative to other enzymes, but also on the baseline degradation rate for Bk in each district [131]. It has been demonstrated that NEP contribution to Bk degradation is higher than ACE in the heart [133,134]. Therefore, it is conceivable that the selective NEP antagonism may increase tissue cardiac Bk levels more than ACE-inhibitors, thereby empowering the local cardioprotective effect of this peptide against MIRI.

### 4.4. Apelin

**Figure 3:**
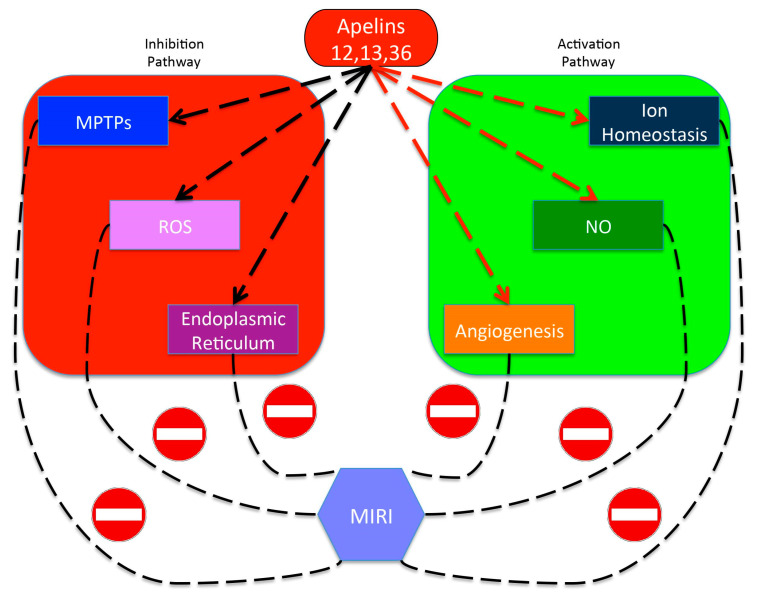
Protective effects of apelins against MIRI. A large body of evidence suggests that apelin-12, -13, and -36 ameliorate MIRI. These apelin isoforms trigger inhibition and activation pathways during MIRI. In particular, they inhibit MPTPs opening, ROS formation, and ER stress. Consistently, they activate ionic channel function for ion homeostasis, NO synthesis, and angiogenesis. All these effects protect the heart against MIRI. MIRI, myocardial ischemia-reperfusion injury; MPTPs, mitochondrial permeability transition pores; ROS, reactive oxygen species; ER, endoplasmic reticulum; NO, nitric oxide.

The pre-pro-apelin consists of 77 amino acid residues and is cleaved into shorter biologically active C-terminal fragments, including apelin-12, -13, -17, and -36 [135]. A large body of evidence suggests that the apelin/APJ system protects the heart against MIRI. In particular, apelin-12, -13, and -36 ameliorate MIRI [136] (Figure 3). Apelin-13 reduces the decline in mitochondrial membrane potential and inhibits the opening of MPTPs by PI3K/Akt/GSK 3β [137]. The administration of apelin-13 and 36 at reperfusion delays MPTPs opening ratios and results in direct cardioprotective actions through PI3K-Akt and p44/42 in MIRI [138]. Besides, activation of PI3K/Akt inhibits the abrupt opening of MPTPs and ameliorates MIRI [139]. Thus, the apelin/APJ system might decrease mitophagy by inhibiting MPTPs opening through PI3K/Akt in MIRI.

Moreover, the apelin/APJ system inhibits the generation of mitochondrial ROS, which leads to the abrupt opening of MPTPs and cell death in response to MIRI [140]. Apelin-12 and apelin-13 selectively inhibit mitochondrial ROS generation and improve heart functional and metabolic recovery after MIRI [141]. Apelin-13 decreases ROS overproduction and oxidative stress-induced apoptosis by PI3K/Akt and MAPK/ERK1/2 signaling pathways under PC [142]. Similarly, apelin-12 and its structural analogue lessen oxidative stress by increasing antioxidant enzyme activation and reducing ROS formation in MIRI [143].

Apelin/APJ system increases NO formation and improves MIRI-induced cardiac dysfunction [144]. The apelin/APJ system promotes NOS activation mainly through enhancing the phosphorylation of Akt. In particular, apelin-12 activates eNOS and induces cardioprotection by PI3K/Akt after reperfusion [145], thereby reducing cardiomyocyte membrane damage, and limiting IS in MIRI through NO formation [146]. Furthermore, NO formation is involved in the process of recovery of energy metabolism induced by the apelin/APJ system. Restoration of energy metabolism increases functional recovery of the heart and decreases cell membrane damage in MIRI [147,148].

The apelin/APJ system plays an important role in angiogenesis [149]. Apelin-13 induces vascular smooth cell proliferation through ERK-dependent [150] and PI3K/Akt signaling pathways [151]. Apelin-13 also promotes capillary density through overexpression of the proangiogenic factor, VEGF, in MIRI [152]. Furthermore, the treatment with apelin-13 significantly increases myocardial capillary density and arteriole formation and improves cardiac repair [153].

Apelin/APJ system may also attenuate endoplasmic reticulum (ER) stress response-induced cell death by promoting NO formation in MIRI [154,155]. The ER is responsible for protein synthesis, folding, maturation, and transport. ER stress not only gives rise to the accumulation of unfolded or misfolded proteins in the ER [156], but also induces cell apoptosis through caspase-12 and JNK [157]. Suppression of ER stress can protect the myocardium from MIRI [158].

Apelin-12 and -13, as well as structural apelin-12 analogues, decrease cell membrane damage through better control of cell membrane integrity and ion homeostasis. Indeed, exogenous apelin-12 significantly decreases LDH leakage and reduces myocardial cell membrane damage by phospholipase-C (PLC), protein kinase C (PKC), PI3K, and MEK1/2 signaling [159]. Inhibition of Na^+^/H^+^ exchange can reverse the effects of apelin-12 on LDH leakage and promote membrane damage in MIRI [159]. Therefore, apelin-12 maintains the integrity of cell membranes through Na^+^/H^+^ exchange.

Finally, the apelin/APJ system significantly affects calcium handling. In isolated rat hearts, apelin-13 improves the redox states of SR Ca^2+^-modulators and maintains Ca^2+^ transient in MIRI [160]. Maintaining of Ca^2+^ transient can inhibit Ca^2+^ transient overload and induce cardioprotection in MIRI. Furthermore, apelin-13 and apelin-36 inhibit reperfusion-induced hypercontracture of heart by delaying the abrupt opening of MPTPs [138].

Despite their fundamental potency, apelin peptides are prone to fast proteolytic degradation [161,162]. So far, ACE-2 [163,164], NEP [87], and human plasma kallikrein (KLKB1) [165] have been identified as metalloproteases that cleave apelins.

### 4.5. Substance P (SP)

SP is an undecapeptide member of the tachykinin neuropeptide family, that acts as a neurotransmitter and as a neuromodulator [166]. SP is released from sensory nerve terminals in both the central and peripheral nervous systems. Although its levels are low in the myocardium, it can affect the heart, due to its role in nociception, inflammation, plasma extravasation, platelet, and leukocyte aggregation in post-capillary venules, and leucocyte chemotactic migration through vessel walls [167]. In the heart, SP-containing nerve fibers are located in the intrinsic ganglia of the myocardium and around coronary vessels. A small number of coronary endothelial cells also contain SP. Of note, localization of SP-containing nerve fibers around the coronary arteries, as well as in coronary artery endothelial cells, may suggest that this neuropeptide is ideally placed to be released in response to changes in coronary arterial pressure or situations of hemodynamic stress. SP can be considered as a potent vasodilator through NO release [168].

SP has two distinct effects in response to myocardial insult or overload. Short-term, SP vasodilatory effects appear to be protective by increasing myocardial perfusion, as demonstrated by ischemia-reperfusion studies. Indeed, SP induces bone marrow stem cell mobilization that has been experimentally and clinically investigated to regenerate damaged hearts and suppresses inflammation in ischemic injuries [169,170,171]. In a murine model of MIRI and acute MI, systemic injection of SP decreased early inflammatory responses and promoted stem cell mobilization, leading to a compact vasculature and improved cardiac function [172]. In particular, it has been reported that SP reduces MIRI-induced cell death by directly acting on cardiac cells to initiate cell survival pathways via the neurokinin receptor 1 (NK-1) and Akt [173].

On the other hand, long-term up-regulation of SP appears to be associated with inflammation, apoptosis, matrix metalloproteinase activation, and alterations of the extracellular matrix, which may collectively lead to adverse remodeling and worsening HF [174].

### 4.6. Adrenomedullin (ADM)

Human ADM is a 52 amino acid peptide, that is produced from its precursor molecule pre-pro-ADM. Since the discovery that the ADM gene is expressed to a greater extent in endothelial cells than in the adrenal medulla, it has been recognized that this peptide should be considered as a secretory product of the vascular endothelium, together with NO and endothelin [175].

The beneficial effects of ADM on MIRI are well-known [176]. It has been demonstrated that ADM gene delivery [177], as well as a short-term infusion of ADM [178], significantly attenuated MIRI in rats. These cardioprotective effects are attributed mainly to the antiapoptotic effects of ADM via a PI3K/Akt-dependent pathway [178]. Moreover, clinical studies showed that ADM reduces pulmonary capillary wedge pressure, increases cardiac index, and increases urinary volume and sodium excretion in patients with HF [179].

Consistently, intermedin (IMD or ADM-2), an ADM similar peptide, plays an important role in MIRI prevention. Administration of IMD attenuates acute IRI in the isolated perfused rodent heart [180,181] and following coronary artery ligation in vivo [182]; this protection is only partly due to IMD-mediated augmentation of coronary blood flow. Direct protective effects of IMD against hypoxia-reoxygenation injury have also been demonstrated in neonatal cardiomyocytes [183].

IMD has been detected in patients without evident cardiac disease post-mortem in left ventricular cardiomyocytes, pericardial adipocytes, pericardial vein endothelial cells, and coronary artery vascular smooth muscle [184]. IMD is expressed more abundantly than ADM in human cardiac microvascular endothelial cells (HCMEC) and cardiac fibroblasts from the human ventricle (v-HCF) [185]. IMD circulates in human plasma at lower levels than ADM, indicative of more limited distribution, but is elevated upon acute MI [186,187]. It has been reported that IMD protects HCMEC and v-HCF in culture against the deleterious effects of simulated acute IRI, predominantly via AM1 receptor involvement [185]. Furthermore, although IMD is present in human cardiomyocytes (HCM), IMD derived from HCMEC and acting in a paracrine manner, predominantly via AM1 receptors, makes a marked contribution to HCM protection by the endogenous peptide against acute IRI [188].

ADM levels in the plasma and tissues are regulated by the proteolysis of NEP, the enzyme mainly involved in ADM degradation [86]. Therefore, NEP inhibition during reperfusion could represent an attractive treatment option in the early management of acute coronary syndrome to salvage myocardial tissue.

## 5. Clinical Perspectives for NEP Inhibition in MIRI Prevention

During the last three decades, timely myocardial reperfusion using either thrombolytic therapy or pPCI has allowed amazing improvements in outcomes with a more than halving in 1-year STEMI mortality [189,190,191]. However, mortality and LVR remain substantial in these patients. Interestingly, LV remodelers are more likely to be admitted to hospital for HF than non-remodelers [6,7]. As such, novel therapeutic interventions are required to reduce IS, preserve LV systolic function, and improve survival in reperfused-STEMI patients.

Actually, MIRI prevention represents the main goal to reach in order to reduce STEMI mortality [192,193]. Nevertheless, the translation of effective cardioprotective strategies tested in animal studies to human treatments has not yet been successful [194]. As an example, while cyclosporine reduced IS in animal models and in preliminary human trials [195], it did not reduce IS in a properly performed randomized clinical trial with a large sample size [196]. Consistently, while metoprolol reduced IS in pigs [197] and in a post hoc analysis of the METOCARD-CNIC (effect of METOprolol of CARDioproteCtioN during an acute myocardial InfarCtion) trial on a little cohort of patients with anterior STEMI [198], it failed to offer any benefit in large clinical trials [199]. Moreover, although Post-C demonstrated marked effects in experimental studies [200], it failed to reduce all-cause mortality and hospitalization for HF in a large trial [201]. Conversely, non-invasive remote ischemic conditioning (RIC; performed during acute ischemia) gave good results in experimental studies [202] and clinical trials with a reduction of cardiovascular events [203,204]. The LIPSIA CONDITIONING trial evaluated the combination of RIC plus Post-C in addition to pPCI [205]. This study showed a significantly increased myocardial salvage assessed by cardiac magnetic resonance imaging and a reduction of new HF incidence compared with conventional pPCI [206]. However, it remains unclear whether cardioprotection was mainly attributable to RIC or to synergistic effects of combining RIC and Post-C, because of the lack of a RIC control group.

An important reason for the weak and inconsistent results obtained in this field may be the presence of multiple, partially redundant, mechanisms of cell death during ischemia-reperfusion, whose relative importance may depend on the conditions. Therefore, it is always more recognized that it is important to consider a “multi-targeted cardioprotective therapy”, defined as an additive or synergistic cardioprotective agents or interventions directed to distinct targets with different timing of application (before, during, or after pPCI) [207].

Given that NEP substrates exert a cardioprotective effect against IRI, it is conceivable that antagonism of proteolytic activity by this enzyme may be considered in a multi-targeted strategy for MIRI prevention. Sacubitril/valsartan (LCZ696; SAC/VAL) is a first-in-class approved angiotensin receptor NEP inhibitor (ARNI), that simultaneously provides NEP inhibition and blockade of the Ang II type I receptors (AT1Rs). This concomitant blocking is required because the single NEP inhibition increases Bk levels leading to clinically relevant episodes of angioedema [208]. SAC/VAL has been successfully used in the treatment of chronic [209] and acute decompensated HF [210]. There are many reasons because SAC/VAL could be an attractive candidate for cardioprotection against MIRI, even if there are essentially no data to support this proposal, so far.

Firstly, concomitant NEP and AT1Rs antagonism increase levels of molecular compounds leading to activation of different pro-survival pathways, such as PI3K-Akt, PKC, PKG, AMPK, and ERK (Figure 1). In particular, augmented Bk levels mediate protection against MIRI at myocardial [125,126,127] end endothelial level [129], whereas AT1Rs blocking inhibits pro-apoptotic pathways mediated by acute Ang II increase. Consistently, the reflex increased Ang II levels may activate cRAS and iRAS that enhance cell protection against oxidative stress [115]. Furthermore, SAC/VAL may prevent the long-term harmful accumulation of SP, because this peptide is broken down by the uninhibited ACE, thereby exploiting acute SP positive effects on MIRI prevention [169,170,171]. NEP is also the main enzyme involved in the degradation of apelins, that are prone to fast proteolysis [161,162]. Therefore, SAC/VAL may significantly increase levels of these potent MIRI antagonists, thereby reducing cardiomyocytes’ necrosis.

As proof of its molecular effects, NEP/AT1Rs inhibition gave good results in different animal experimental models of MI [211,212,213]. In fact, it has been demonstrated that SAC/VAL prevents cardiac rupture in a murine model of MI [214] and plays a pro-survival effect in reperfused rabbit hearts after prolonged ischemia [215].

The timing of SAC/VAL therapy is also important. Following oral administration, SAC/VAL dissociates into individual components with plasma concentrations of SAC, its active metabolite sacubitrilat (LBQ657), and VAL achieving peaks in 0.5 h, 2 h, and 1.5 h, respectively. In clinical study PIONEER-HF, the time-averaged reduction of the N-terminal pro-B-type natriuretic peptide (NT-proBNP) concentration was significantly greater in the SAC/VAL group than in the enalapril group. Interestingly, the greater reduction in the NT-proBNP concentration with SAC/VAL than with enalapril was evident as early as week 1 (ratio of change, 0.76; 95% CI, 0.69 to 0.85) [210]. Furthermore, considering urinary cyclic guanosine 3′-5′ monophosphate (ucGMP) as a measure of the biological effect of SAC/VAL on NP-mediated activation of NP receptors, serial measurement of ucGMP revealed a very significant increased concentration in patients treated with SAC/VAL compared with a decline in ucGMP concentration in the enalapril group (*p* < 0.001 for SAC/VAL versus enalapril at 1 week through 8 weeks) [216]. Thus, SAC/VAL shows a very fast action on the increase of NPs levels that could allow an earlier cardioprotective effect against MIRI.

Given these findings, in a “multi-targeted” strategy aimed at MIRI prevention, we hypothesize that optimal timing for SAC/VAL may be immediately at the end of pPCI, after RIC protocol administration and instead of Post-C. In fact, RIC has been shown to reduce the incidence of major adverse cardiac events, in particular of HF, whereas the role of Post-C has been not well defined [206]. In this context, SAC/VAL might prolong the window of cardioprotection induced by RIC, thereby more efficiently antagonizing MIRI.

Interestingly, SAC/VAL is not in contrast with other drugs commonly used in STEMI treatment. Dedicated drug interaction studies demonstrated that co-administration of furosemide, carvedilol, amlodipine, omeprazole, hydrochlorothiazide, metformin, and atorvastatin, did not alter the systemic exposure to SAC, sacubitrilat, or VAL [217]. Nevertheless, we have to keep in mind that there are also some limitations to SAC/VAL use in patients affected by acute MI. As an example, hypotension is a very frequent potential collateral effect of this therapy, and SAC/VAL has not to be used if blood systolic pressure is below 100 mmHg [217]. SAC/VAL can be titrated from the lowest to the highest dosage according to blood pressure values in order to reduce the risk of hypotension [218]. Furthermore, concomitant administration of potassium-sparing diuretics (e.g., the mineralocorticoid receptor antagonist spironolactone, also used as an anti-ventricular remodeling agent) [219], potassium supplements, or salt substitutes containing potassium may lead to an increase in serum potassium concentrations. In patients who are elderly, volume-depleted (including those on diuretic therapy), or with compromised renal function, SAC/VAL may result in worsening of renal function, including possible acute renal failure [217].

It is hoped that most doubts about SAC/VAL utilization in the setting of acute MI may be clarified by the results of the ongoing PARADISE MI study (NCT02924727). Nevertheless, it is noteworthy to mention that the benefits of SAC/VAL on post-MI LV remodeling may be mitigated on the background of optimal medical therapy (beta-blockers, mineralocorticoid receptor antagonists, previous treatment with RAAS inhibitors). Consistently, sodium-glucose transport protein 2 (SGLT2) inhibitors have been shown to ameliorate post-MI LV remodeling in a porcine model of MI [220]. Thus, it is entirely possible that a future more extensive administration of these drugs in patients affected by STEMI may attenuate the benefits of SAC/VAL.

## 6. Conclusions

In STEMI, coronary reperfusion itself induces myocardial injury that can be prevented by endogenous compounds targeting different pro-survival pathways. These molecules are the substrate of various peptidases that regulate their plasmatic levels. NEP plays a pivotal role in enzymatic degradation of most of them (NPs, Ang, Bk, apelin, SP, and ADM), especially in the setting of acute MI. Thus, it is conceivable that NEP inhibition could represent an interesting target to prevent MIRI. SAC/VAL is a well-known ARNI yet successfully used in the treatment of chronic and acute decompensated HF. Because of its concomitant selective inhibitory action on NEP and RAS, SAC/VAL has a wide protective effect on myocardium in experimental models of MI. This is currently under investigation for cardiovascular protection against MIRI in humans (PARADISE MI study).
